# Damage Characterization during Compression in a Perlite-Aluminum Syntactic Foam

**DOI:** 10.3390/ma12203342

**Published:** 2019-10-14

**Authors:** Csilla Kádár, František Chmelík, Dávid Ugi, Kristián Máthis, Michal Knapek

**Affiliations:** 1 Department of Materials Physics, Eötvös Loránd University, Pázmány P. stny. 1/A, H-1117 Budapest, Hungary; ugdtaat@caesar.elte.hu; 2 Department of Materials Science and Engineering, Faculty of Mechanical Engineering, Budapest University of Technology and Economics, Műegyetem rkp. 3., H-1111 Budapest, Hungary; 3 MTA–BME Lendület Composite Metal Foams Research Group, Műegyetem rakpart 3., H-1111 Budapest, Hungary; 4 Department of Physics of Materials, Charles University, Ke Karlovu 5, CZ12116 Prague 2, Czech Republic, , knapek@karlov.mff.cuni.cz (M.K.); 5 Nuclear Physics Institute, The Czech Academy of Sciences, Řež 130, 250 68 Řež, Czech Republic

**Keywords:** metal matrix composite, failure mechanisms, mechanical properties, acoustic emission

## Abstract

Aluminum matrix (Al99.5) syntactic foam containing expanded perlite particles was produced using the pressure infiltration technique. The dominant deformation mechanisms during compression of this foam were determined by sequential k-means analysis of the acoustic emission data. Since the different deformation mechanisms were concurrently active even at small strains, successive unloading and reloading measurement was proposed for cluster identification. The repetitive unloading and reloading allowed us to identify two mechanical parameters, namely the unloading modulus and the loss for unloading-reloading cycles. Based on the correlations among the strain localization within the specimen, the acoustic emission results, the changes in these mechanical parameters, and the transition from quasi-elastic deformation to plasticity were revealed in this material.

## 1. Introduction

Metal matrix syntactic foams (MMSFs) are extensively studied as novel materials [1,2,3,4]. They show great potential for energy absorption applications [1], and since MMSFs are stiff, light, and fire-retardant, they can be also used for structural applications, such as lightweight cores for panels and tubes. In this respect, monitoring the deterioration of the material (i.e., monitoring the accumulation of the failure occurring during the deformation) is particularly important. 

In recent years, the effects of different macroscopic and microscopic parameters on the mechanical properties of perlite particles-filled aluminum syntactic foams have been extensively investigated [5,6,7,8,9,10,11,12]. These foams were found to be a good replacement for conventional metal foams since they exhibit low density (0.75–1.1 g/cm^3^) and high energy absorption efficiency [10]; furthermore, the structure and the density of syntactic foams can be easily adjusted to meet the requirements for particular application. Moreover, the production of perlite-aluminum syntactic foam is cost-effective as the pressure infiltration process involved in the foam production is inexpensive and the expanded perlite (EP) is likewise cheap [13]. 

The other advantage of the pressure infiltration process is that it results in an isotropic structure. Manufacturing processes can introduce anisotropy to the cell structure (e.g., freeze casting and GASAR method) that results in direction sensitivity of mechanical properties. This phenomenon is well-known and widely investigated in the literature [14,15]. However, in the case of pressure infiltration process, if the incorporated particles show isotropic properties, the resultant MMSFs were found to show isotropic behavior [5].

The investigations have so far revealed that the foam density [5,10], the shape and the size of the EP particles [8,11], and the properties of the metal matrix [6] are the dominant parameters that primarily determine the compressive behavior of the perlite-aluminum syntactic foam [6]. This is consistent with the behavior of conventional foams found by Ashby and Gibson [16], which can be described as: $X^{*}\;=\;X\cdot \beta \cdot \rho_{rel}{}^{n}$, where a given property of the foam and the same property of the material it is made of are X^*^ and *X*, respectively, *β* and *n* are constants related to the shape and the character (closed or open cells) of the cells, respectively, and $\rho_{rel}$ is the relative density (the ratio of the foam’s density and the density of the bulk material it is made of). This similarity is given by the fact that EP particles exhibit practically no strengthening effect [7]. 

It was further reported that the deformation mechanisms can be influenced by changes in the foam density [5] or local density variations [10]. It was found that perlite-aluminum syntactic foam with A356 aluminum alloy matrix and EP particles of 3–4 mm in diameter deforms uniformly with multiple active collapse bands if the density is higher than approximately 1.09 g/cm^3^. In samples with lower density, the deformation is localized in large individual shear bands at an angle of approximately 45°–65° with respect to the compression axis [5]. 

The deformation mechanisms and failure during compression can be effectively investigated by means of acoustic emission (AE). AE represents transient elastic waves that originate within the material due to sudden localized structure changes, such as collective dislocation motion or crack initiation and propagation. Thus, AE yields information on the deformation mechanisms during the deformation of the material. It has been proven that applying the recently developed adaptive sequential k-means (ASK) clustering method on the raw AE data allows separating AE events originating from different sources (i.e., different deformation mechanisms). In this method, the AE signal is first sectioned into time-windows. Clustering is based on the statistical parameters of power spectral density functions (like energy and mean frequency), which are then determined for each time-window. For details on the algorithm, see Reference [17]. However, in EP aluminum syntactic foams, many deformation mechanisms can be concurrently active (e.g., plastic deformation and failure of the matrix material, and collapse of perlite particles), which makes the AE analysis rather challenging.

The aim of this work was to find mechanical parameters that can help predict the progress of failure in EP syntactic foams. These foams are particularly a good choice for this kind of investigations since the EP particles have low strength and they are brittle, so that the matrix material deforms the particles crumble, enhancing the failure progress. Successive unloading and reloading measurements were proposed to monitor the evolution of failure in the EP syntactic foam since both the unloading modulus and the area of the hysteresis loops of the stress-strain curve (i.e., mechanical work) can characterize the failure state of a syntactic foam [18,19]. Furthermore, the repetitive loading-unloading testing is also advantageous for the above-mentioned ASK evaluation of AE signals since during unloading some mechanisms generating AE become inactive, thus helping to properly identify the particular AE sources in the AE frequency spectra.

## 2. Materials and Methods 

The perlite-aluminum syntactic foam was produced by the pressure infiltration technique at the Eötvös Loránd University, Budapest. First, EP particles (with compositions of 72 wt% SiO_2_, 12 wt% Al_2_O_3_, 3 wt% Na_2_O, 4 wt% K_2_O, 2 wt% CaO, and 1 wt% MgO as the main constituents, provided by the supplier) with a diameter between 1 and 2 mm were poured into a closed-profile crucible with a cross-section area of 50 mm × 50 mm and were slightly compacted. Thereafter, a stainless steel mesh was fixed on top of the particles to avoid the movement of the EP particles during the infiltration. A block of commercially pure Al (Al99.5) was placed on the mesh and the closed-profile crucible was enclosed by welding. During heating, the interior of the closed-profile crucible was kept in helium atmosphere using an inlet, which was welded to the closed-profile crucible. The assembly was heated up to 750 °C and held at this temperature for 30 min. Then, 1 bar overpressure of helium gas was applied for 2 min to complete the infiltration. Finally, the crucible with the infiltrated EP particles was quenched into water at ambient temperature. Skin with a diameter of 2 mm was removed at the sides and the bottom of the syntactic foam block. 

The uniaxial compression tests with repetitive loading-unloading cycles were carried out with a custom-made mechanical testing machine in displacement control at an initial strain rate of 0.007 s^−1^ at room temperature. Three cube-shaped foam specimens with a side length of 13.5 mm were tested. The surface skin incurred during the sample preparation was removed by mechanical polishing. The density was determined as the quotient of the mass and the product of the length of the foam block. The obtained density was around 1.1 g/cm^3^. Teflon strips were used to minimize the friction effect between the sample and the grips. In order to minimalize the effect of the testing machine on the data, the displacement was measured by an extensometer clipped on the grips. The surface displacement during straining was monitored by a high-resolution video camera. 

The AE response was recorded during straining using the Vallen AMSY-6 system [20]. The AE signal was stored in transient recording mode, i.e., the raw signal was recorded. The sampling rate was 1 MHz, without using a threshold level. The AE signal from the sensor was pre-amplified using the Vallen pre-amplifier AEP5H (Vallen Systeme GmbH, Icking, Germany) with a gain of 40 dB. A WSα-type sensor produced by Physical Acoustics Corporation was coupled to the specimen surface with vacuum grease and an elastic band. 

Based on the high-speed camera recordings, areas of interest during compression were identified on the specimen surface and further examined by a Hitachi TM4000 Tabletop scanning electron microscope (Tokyo, Japan). Slices for the microscopy were cut from the deformed sample with a diamond disc cutter at a low speed to minimize the damage of the PE particles. In order to reveal the strain distribution during deformation, a digital image correlation (DIC) analysis was performed using the Ncorr scripts implemented in Matlab [21]. 

## 3. Results

**Figure 1:**
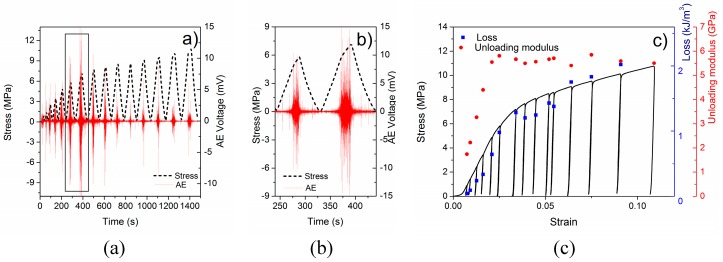
(**a**) The stress during loading and unloading as a function of time (dashed black line) and the corresponding acoustic emission response (solid red line) of an expanded perlite-aluminum syntactic foam; (**b**) the stress during the 6^th^ and 7^th^ cycles as a function of time (dashed black line) and the corresponding acoustic emission response (solid red line) of an expanded perlite-aluminum syntactic foam; (**c**) a typical stress-strain curve (black line) and the unloading modulus (red dots) and loss (blue squares) at given strains.

A typical time-stress curve with an AE response of an EP aluminum syntactic foam during repetitive loading and unloading and the corresponding deformation curve can be seen in Figure 1a,c, respectively. (Figure 1b shows the AE response during the 6^th^ and 7^th^ cycles of loading and unloading.) The compression tests were stopped at 2200 N which was the force limit of the testing machine. In this way, we monitored the damage mechanisms at low strains (<12%). In this deformation range, the stress-strain curve consists of two stages: a quasi-linear stage and a plastic region. In order to avoid recording of spurious AE signals originating from the machine at the strain direction reversal occurring at zero stress, the unloading was terminated approximately at 200 N.

The AE activity was observed practically as soon as the compression started. In the quasi-linear stage, the AE activity increases with increasing stress and it reaches its peak intensity approximately at the end of the quasi-linear stage. This behavior is similar to that of other aluminum matrix syntactic foams [22,23]. Even during unloading and reloading periods, the AE activity was observed (Figure 1b). Within one cycle, the AE activity exhibits its minimum value at the end of unloading and at the beginning of reloading and, subsequently, it increases with increasing stress (Figure 1a,b). As soon as the stress approaches the maximum value reached in the previous cycle, there is a rapid increase in the AE activity even in the quasi-linear stage. It is worth noting that as the unloading starts the AE activity drops immediately, but it still remains well above the noise level (Figure 1b). 

The loss during an unloading and reloading cycle ($\oint_{\varepsilon_{unloadig,\;reloading}}\sigma d\varepsilon$) and the unloading modulus (the slope of the stress-strain curve at the beginning of unloading) of the foam for each cycle were also determined (Figure 1c). The loss is negligible (<10 Jm^−3^) at the beginning of the deformation (since the unloading and reloading curves are almost identical) and it increases with increasing applied strain. We found that the rate of the rise of the unloading modulus changes and the loss value changes during deformation (Figure 1c). 

**Figure 2:**
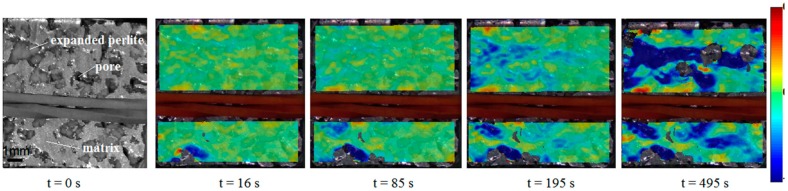
The original image of the sample and the deformation of the surface in the direction parallel to loading at t = 16 s (ε = 0.006), t = 85 s (ε = 0.013), t = 195 s (ε = 0.021), and t = 495 s (ε = 0.04).

The surface strain mapping resulting from the DIC analysis of the video revealed that even at small strains plastic deformation or even fracture can occur due to stress localization (Figure 2, see also Supplementary Materials, Video S1, where the fracture of the foam is visible on the surface at 16 s). Upon further compression, at ~90 s, a small deformation zone at an angle of approximately 45° in the left bottom corner of the sample appears, while at ~190 s the formation of a deformation band starts in the upper part of the foam. At ~490 s, the collapse of the cells in an almost horizontal deformation band is discernible.

**Figure 3:**
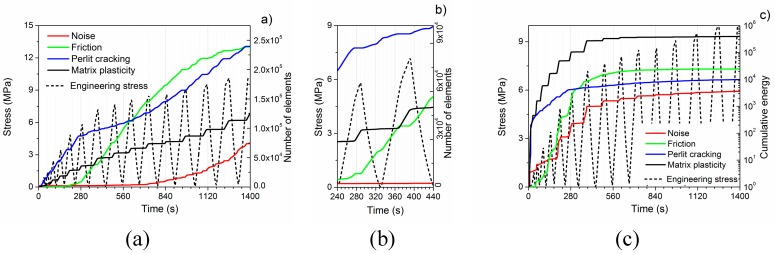
(**a**) Time evolution of the cumulative number of elements; (**b**) the cumulative number of elements during the 6^th^ and 7^th^ cycles; and (**c**) the cumulative AE energy in the AE clusters assigned to the noise (red line), matrix plasticity (black line), friction between the fractured falls of EP particles (green line), and the cracking of EP particles (blue line). The deformation curve is displayed by the dashed black line.

In order to identify the failure mechanisms during deformation, the AE signal was analyzed using the ASK procedure. The ASK evaluation revealed four clusters. The time evolution of the cumulative number of the elements and the cumulative AE energy of each cluster can be seen in Figure 3. The following dominant source mechanisms were assigned to the clusters.

• Cluster 1—Noise (color code: red)

**Figure 4:**
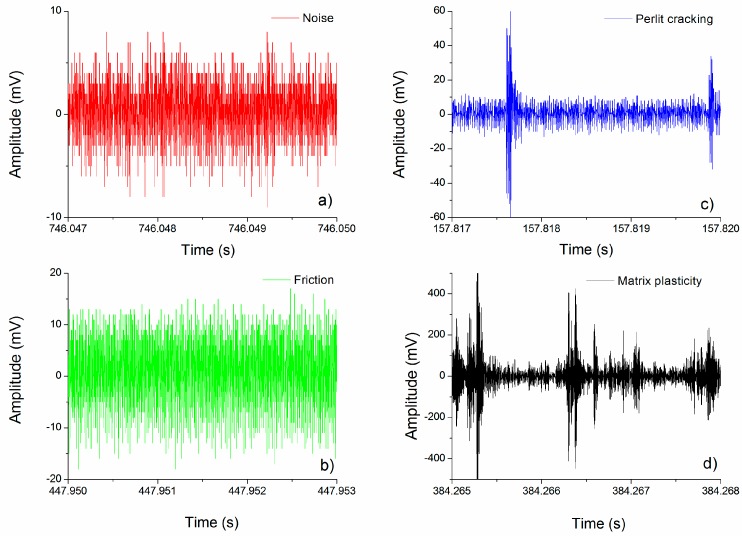
Characteristic waveforms in the particular clusters: (**a**) noise (red), (**b**) friction (blue), (**c**) expanded perlite particle cracking (blue), and (**d**) matrix deformation (black).

Since the AE measurement was launched before starting the deformation test, the first cluster naturally belongs to the noise. This cluster mostly consists of AE signals with low amplitude (cf. Figure 4a). The significant increment in the number of these elements after 500 s suggests that the other mechanisms do not produce a considerable AE activity anymore.

• Cluster 2—Friction inside the syntactic foam (color code: green)

This cluster is active during both unloading and reloading. It is not active in the quasi-linear stage; however, it becomes active as the formation of the deformation band begins (Figure 2 and Video S1 in Supplementary Materials). The AE response in this cluster is similar to that in cluster 1. The observed sequence of low-amplitude, continuous-like signals (Figure 4b) well corresponds to the observations of other researchers [24].

• Cluster 3—Cracking of EP (color code: blue)

Cracking of EP particles is the most dominant AE source in the quasi-linear stage. The burst character of the signals with a short rise time is typical of fracture (Figure 4c). The burst signals have low energy, which can be attributed to the thin walls of the EP particles.

• Cluster 4—Plastic deformation and crack initiation and propagation in the matrix (color code: black)

Cluster 4 is active only during reloading when the stress exceeds the maximum stress value of the previous cycle (Figure 3a,b). This cluster gives the most significant contribution to the cumulative energy during the formation of the first large deformation band (see Video S1 at around 380 s in Supplementary Materials). The bending and the fracture of cell walls in the deformation band are well visible on the video, which suggests that the source of cluster 4 is plastic deformation and crack initiation and propagation in the matrix material. This is supported by the fact that AE signals in this cluster are typical of collective dislocation movement (Figure 4d) [25].

## 4. Discussion

**Figure 5:**
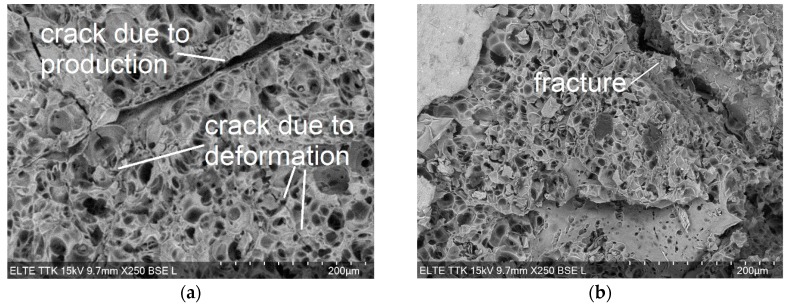
SEM images of the perlite in a deformed area with the section perpendicular to the direction of loading shown: (**a**) cracks of different origins; (**b**) a crack due to fracture.

The ASK analysis revealed that, even at small stress, the matrix of the EP aluminum syntactic foam yield and, simultaneously, the cell walls of the EP particles are being crushed. To confirm the EP cracking as a possible AE source, sections of deformed samples were investigated by SEM. The deformed sample was compressed at the same conditions as other samples. The compression was stopped at 1 MPa in the quasi-elastic stage (the stress at the end of the quasi-linear stage is about 5 MPa). The inspection of the SEM images of the deformed samples showed that in the EP particles two types of cracks can be distinguished. One of them is straight, canyon-like and can be seen on the SEM images of both the deformed and undeformed foam and originates from the foam production [5]. The second crack type, which results from the deformation of the EP particles, is jagged and can be found only in the deformed sample (Figure 5). This observation agrees with results found in Reference [7], i.e., that the EP deforms even at low stresses and has practically no strengthening effect.

**Figure 6:**
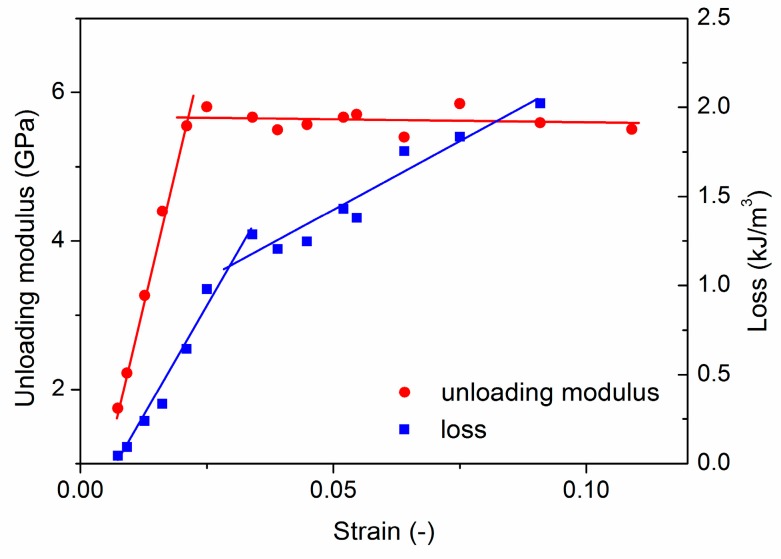
The unloading modulus (in red) and the loss (in blue) as a function of strain.

The changes in the unloading modulus as a function of strain can be seen in Figure 6 (the unloading modulus was not determined for the first unloading since there were not enough points for the fitting in the unloading part of the stress-strain curve). It is clear that there is a significant change in the slope of the unloading modulus evolution approximately at the end of the quasi-linear stage (Figure 1c and Figure 6). It should be noted that we found similar trends in the expanded clay syntactic foams. We suppose that the changes in the unloading modulus are a consequence of the presence of micropores, namely, during infiltration, micropores are formed between EP particles that are very close to each other. Due to stress localization, the foam starts to deform around these micropores causing the closure of them. As the deformation proceeds, the strain contribution due to the closure of the pores decreases, resulting in an increase in the unloading modulus. 

Figure 6 shows the changes in the loss as a function of strain. The changes in the hysteresis area were also observed in References [18,26]; however, the area was not determined in these cases. We found that there is a change in the slope of loss values, similar to that in the unloading modulus. At the strain of ~3% where the slope changes, the number of elements in cluster 2 (friction) starts to increase (Figure 3b), suggesting that loss is related to the friction in the foam. Video S1 (Supplementary Materials) also confirms that after the quasi-linear stage, formation of the deformation band starts, and the EP particles start to crash causing a sudden increase in the number of broken EP cell walls. Taking this into consideration, we can assume that the increase in the loss can be connected with the increase in the number of broken EP particle cell walls, and since the EP particles have no strengthening effect, the increase in the loss reflects not only the damage of the EP particles but also the damage of the matrix. 

The AE measurements revealed that plastic deformation takes place already during the quasi-linear stage, but both the cumulative energy and the number of element in cluster 4 (matrix plasticity) are much lower than in the plastic stage. This means that the quasi-linear stage can be characterized mostly by microplasticity, i.e., only a small volume suffers plastic deformation, while in the plastic stage macroplasticity occurs. This is clearly visible both on the deformation of the surface (deformation starts to localize at around 200 s (4^th^ unloading), Figure 2) and in the AE response (the AE activity is increased to ~150% at the end of the quasi-linear stage (Figure 1a) and also the energy in the cluster 4 (matrix deformation) increases rapidly (Figure 3c)). This allows us to identify the end of the quasi-linear stage as the starting point of the failure accumulation. We found that changes in the rise of both the unloading modulus and the loss occur at the beginning of the failure accumulation and, therefore, they are able to characterize the failure state of a syntactic foam. 

## 5. Conclusions

The failure mechanisms of an EP aluminum syntactic foam during compression up to 12% strain were investigated by AE and by defined mechanical parameters. The ASK analysis was performed on the AE data in order to determine the different deformation mechanisms on a microscopic level.

(1) We identified three main failure processes during the compression of an EP aluminum syntactic foam: plastic deformation of the matrix material, and cracking of walls of the EP particle and friction of these broken walls. These mechanisms start almost at the same time at very low stresses; however, the dominant deformation mechanism changes with the deformation. First, the cracking of the EP is the dominant deformation mechanism. At the end of the quasi-linear stage, the formation of a deformation band starts and, as a consequence, the effect of friction becomes more significant.
(2) We found that the end of the quasi-linear stage and the beginning of macroplasticity can be indicated by two mechanical parameters, namely the unloading modulus and the area of the stress-strain curve upon unloading and reloading (loss). The changes in these parameters predict the changes in failure mechanism.

## Supplementary Materials

The following are available online at https://www.mdpi.com/1996-1944/12/20/3342/s1, Video S1: perlite_aluminum_foam.mp4.
